# Effects of triclosan on bacterial community composition and *Vibrio* populations in natural seawater microcosms

**DOI:** 10.1525/elementa.141

**Published:** 2017

**Authors:** Keri Ann Lydon, Donna A. Glinski, Jason R. Westrich, W. Matthew Henderson, Erin K. Lipp

**Affiliations:** * Department of Environmental Health Science, University of Georgia, Athens, Georgia, US; † Oak Ridge Institute of Science and Education, U.S. Environmental Protection Agency, Athens, Georgia, US; ‡ U.S. Environmental Protection Agency, Office of Research and Development, NERL/EMMD, Athens, Georgia, US

**Keywords:** Triclosan, Vibrio, Emerging Contaminant, Coastal, Resistance

## Abstract

Pharmaceuticals and personal care products, including antimicrobials, can be found at trace levels in treated wastewater effluent. Impacts of chemical contaminants on coastal aquatic microbial community structure and pathogen abundance are unknown despite the potential for selection through antimicrobial resistance. In particular, *Vibrio*, a marine bacterial genus that includes several human pathogens, displays resistance to the ubiquitous antimicrobial compound triclosan. Here we demonstrated through use of natural seawater microcosms that triclosan (at a concentration of ~5 ppm) can induce a significant *Vibrio* growth response (68–1,700 fold increases) in comparison with no treatment controls for three distinct coastal ecosystems: Looe Key Reef (Florida Keys National Marine Sanctuary), Doctors Arm Canal (Big Pine Key, FL), and Clam Bank Landing (North Inlet Estuary, Georgetown, SC). Additionally, microbial community analysis by 16 S rRNA gene sequencing for Looe Key Reef showed distinct changes in microbial community structure with exposure to 5 ppm triclosan, with increases observed in the relative abundance of Vibrionaceae (17-fold), Pseudoalteromonadaceae (65-fold), Alteromonadaceae (108-fold), Colwelliaceae (430-fold), and Oceanospirillaceae (1,494-fold). While the triclosan doses tested were above concentrations typically observed in coastal surface waters, results identify bacterial families that are potentially resistant to triclosan and/or adapted to use triclosan as a carbon source. The results further suggest the potential for selection of *Vibrio* in coastal environments, especially sediments, where triclosan may accumulate at high levels.

## Introduction

Worldwide, coastal areas are increasingly impacted by growing population pressures (Crosset et al., 2004). In particular, increasing rates of wastewater discharge are affecting coastal water quality, typically noted through the introduction of enteric pathogens and nutrients (Mallin et al., 2000). Additionally, even properly treated wastewater can discharge chemical contaminants of emerging concern, including pharmaceuticals and personal care products (PPCPs) (Kolpin et al., 2002; Ellis, 2006; Hedgespeth et al., 2012; Du et al., 2014). PPCPs have potentially toxic effects on fish and invertebrates in impacted waters (Kim et al., 2009; Ramirez et al., 2009; Overturf et al., 2015), but despite the ubiquity of antimicrobials in PPCPs, few studies have examined their effects s pecifically on microbial communities (Peele et al., 1981; Grimes et al., 1984; Guerra et al., 2014). Research to date, which has focused on biofilms in freshwater systems, suggests that PPCPs can alter the composition of natural bacterial communities (Yergeau et al., 2010; Lawrence et al., 2012; Yergeau et al., 2012); however, there is a dearth of information regarding PPCP impacts in brackish and marine waters. Furthermore, the effects of PPCPs and associated antimicrobials on naturally occurring pathogens in aquatic systems are largely unknown.

Among the most notable of naturally occurring aquatic pathogens are members of the genus *Vibrio*, which are primarily found in coastal and marine environments. Rates of reported human *Vibrio* infections have risen significantly in recent years both globally and in the United States (Newton et al., 2012). The habitat for these pathogens is expanding due in part to rising sea surface temperatures (Newton et al., 2012; Vezzulli et al., 2012; Baker-Austin et al., 2013; Jacobs et al., 2015; Vezzulli et al., 2016). Additionally, at local scales *Vibrio* can shift from a minor component of the marine bacterial community to a dominant member over short time frames arising from a variety of discrete conditions and significantly altering bacterial community composition (Takemura et al., 2014; Vezzulli et al., 2016; Westrich et al., 2016). Furthermore, antibacterial resistance, which is well-documented among *Vibrio*, can also regulate ecological population structure in marine habitats (Cordero et al., 2012). To date, the effects of PPCPs and associated antimicrobials on the population dynamics of *Vibrio* in environmental communities has not been addressed and could be a novel factor contributing to short-term population expansion or increased antimicrobial resistance.

The antibacterial additive triclosan (2,4,4’-tricloro-2’– hydroxydiphenyl ether) has been a common ingredient in hand soap, toothpaste, sunscreen, and numerous other personal care products and is one of the most ubiquitous antimicrobial compounds found in PPCPs entering surface waters in the U.S. (Kolpin et al., 2002; Sabaliunas et al., 2003; Yazdankhah et al., 2006; Arpin-Pont et al., 2016). Although recently banned in hand soap in the U.S. (U.S. Food and Drug Administration, HHS 2016), triclosan is still in use in other personal care products and can be measured in sediment cores dating back 30 years (Singer et al., 2002; Miller et al., 2008). Early research reported that at least one strain of *Vibrio cholerae* was 20 times more resistant to triclosan than *E. coli*, due to the presence of FabV, a triclosan-resistant isoform of the FabI fatty acid biosynthesis protein, which is a target for triclosan (Massengo-Tiassé and Cronan, 2008). More recent work suggests that resistance may be common across the *Vibrio* genus in both environmental and clinical strains (DeLorenzo et al., 2014).

The main objective of this study was to analyze the impact of triclosan on marine bacterial communities and specifically *Vibrio* population dynamics. We hypothesized that relative *Vibrio* growth would increase in natural seawater microcosms exposed to triclosan through selection of resistant *Vibrio* bacteria.

## Materials and methods

### Experimental conditions

**Figure 1:**
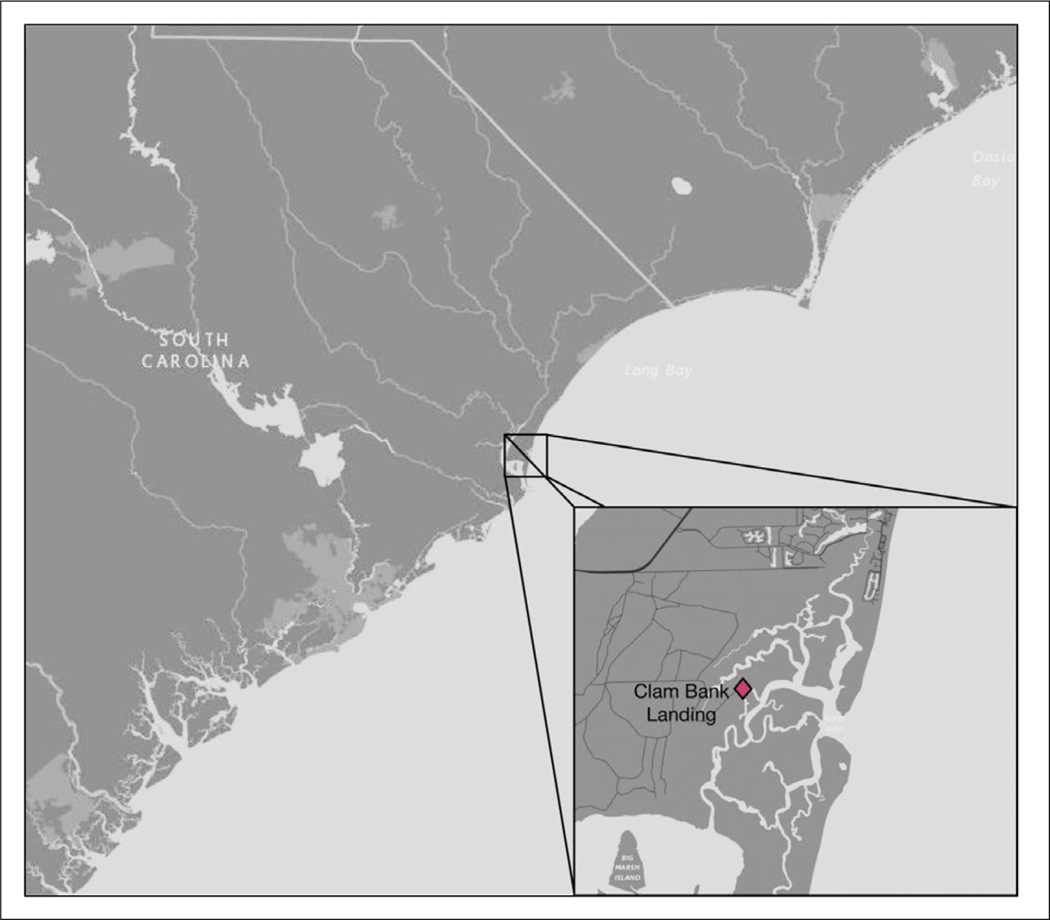
Map of North Inlet Estuary, Georgetown, SC, USA. Sampling site Clam Bank Landing indicated by diamond. DOI: https://doi.org/10.1525/elementa.141.f1

**Figure 2:**
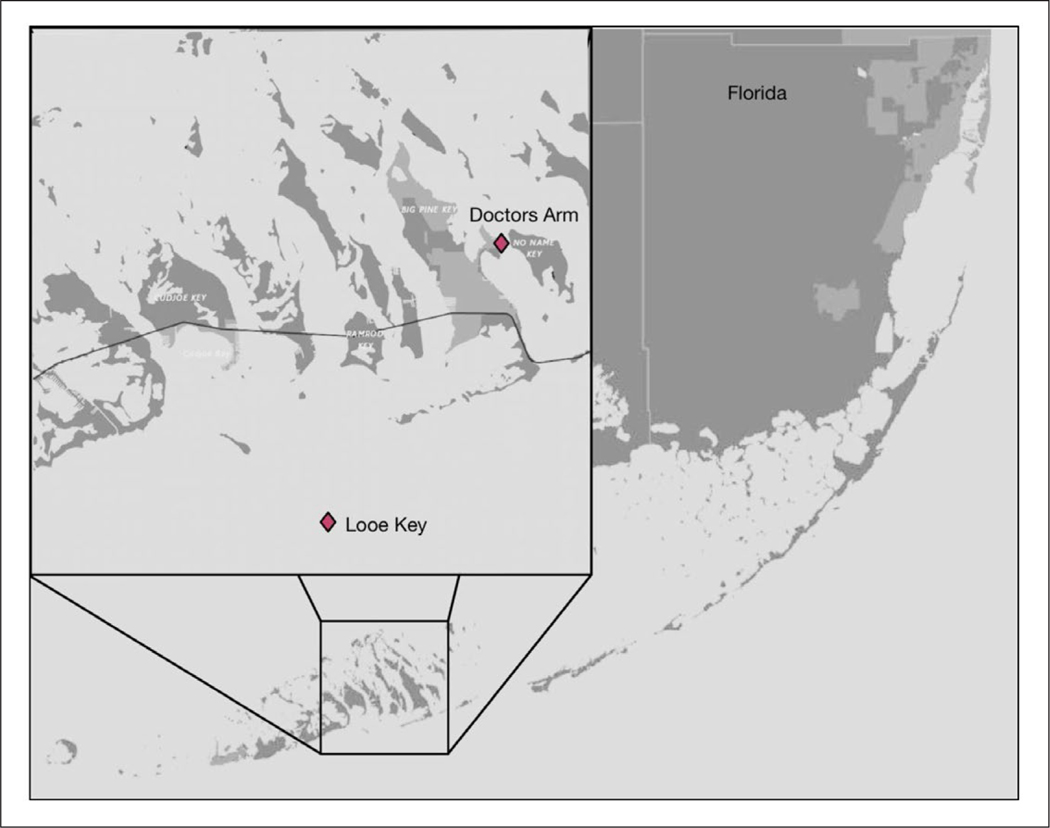
Map of FL Keys, USA. Sampling sites Doctors Arm Canal (Big Pine Key, FL) and Looe Key Reef indicated by diamonds. DOI: https://doi.org/10.1525/elementa.141.f2

Controlled microcosms were used to evaluate the effects of triclosan addition on bacterial communities in natural seawater from three distinct coastal sites with different potential human waste impacts. These included an estuarine location within the North Inlet Estuary, Georgetown, SC (Clam Bank Landing; 33.333933 N, 79.192913 W), with very little immediate development or direct human impact (Figure 1). Two other stations were located in the Florida Keys. A residential canal in Big Pine Key, FL (Doctors Arm; 24.700294 N, 81.351825 W) was highly impacted by s eptic systems and has been previously shown to have consistently high levels of human fecal contamination (Figure 2; Griffin et al., 1999). The final station was located >5 km offshore in oligotrophic waters of the Florida Keys Marine Sanctuary at Looe Key Reef (24.5449 N, 81.40713 W), a popular destination for diving and snorkeling (Figure 2; Szmant and Forrester, 1996).

Experiments were conducted in August 2014. At each station, surface water samples (from <1 m depth) were collected in 15 autoclave-sterilized 1-L polypropylene bottles and experiments were initiated within 3 h of collection. For each station, three replicate bottles were randomly selected and assigned as time zero (T_0_) and sampled immediately. The remaining 12 bottles were assigned to different triclosan treatments, in triplicate. These treatments included no-triclosan addition (no treatment control), solvent control (0.05% ethanol), low triclosan (final target concentration of 5 μg L^–1^ [5 ppb]), and high triclosan (final target concentration of 5,000 μg L^–1^ [5 ppm]). Concentrations were chosen based on previously reported triclosan levels in surface waters (Bedoux et al., 2012) and sediments (Miller et al., 2008; SCCS, 2010). Triclosan working stocks (10 mg mL^–1^) were prepared by dissolving triclosan (Irgasan, ≥97.0% HPLC, Sigma Aldrich) into 100% ethanol (Decon Laboratories).

All microcosm bottles, except for T_0_, were placed in a running seawater raceway in natural light, with neutral density shade cover for 24 h to establish conditions as close to ambient as possible. Experiments were conducted at the Mote Tropical Research Lab (Summerland Key, FL) for Doctors Arm and Looe Key Reef and at the Baruch Marine Field Laboratory (Georgetown, SC) for Clam Bank Landing. An additional set of microcosm experiments was conducted at Clam Bank Landing to evaluate the possible top-down effects of grazers exposed to triclosan (Figure S1). Temperature was measured at time of collection and HOBO pendant loggers (Onset Computer Corporation, Bourne, MA) were used to record temperature in the raceways at hourly intervals. At all collection points, samples were analyzed for total culturable *Vibrio* and triclosan concentrations. For experiments conducted at Looe Key Reef, additional samples were processed for microbial community composition, *Vibrio* cell counts (cell equivalents using qPCR), and total bacterial cell counts (qPCR), as described below.

### Triclosan analysis

Prior to experimental additions of triclosan, 50 mL aliquots were collected from each replicate microcosm bottle and stored in polypropylene tubes in the dark at –20°C for analysis of pre-seed (background) triclosan levels at each station. We found that storage in polypropylene versus amber glass bottles had no effect on triclosan measurements (Supplementary material; Table S1). Within 30 min of each triclosan addition, 50 mL aliquots were removed and held at –20°C to confirm concentration of triclosan additions. All 50 mL water samples were passed through a preconditioned solid phase extraction (SPE) cartridge (Oasis HLB 3 cc 60 mg) at a rate of 10 mL min^–1^. Sample containers were rinsed with 5 mL of Milli-Q water (18.2 MΩ * cm) and added to the SPE cartridge. All cartridges were dried under vacuum for 45 min and eluted with 6 mL of methanol (MeOH) into a 20 mL glass test tube. Methanol fractions were blown down to dryness under a steady stream of nitrogen and reconstituted in 1 mL of 30% MeOH:H_2_O and analyzed via LC-MS/MS.

Triclosan was quantified on a Thermo Accela HPLC coupled to a Thermo Quantum AM quadrupole mass spectrometer equipped with an Eclipse XDB-C18 column (3.5 μm particle size, 3.0 × 150 mm, Agilent Technologies, CA, USA). Initial conditions were held for 0.5 min at 70% water with 0.1% formic acid (A) and 30% acetonitrile with 0.1% formic acid (B), then ramped to 95% B over 14.5 min and held for 1 min before returning to starting conditions and equilibrating for 8 min. Flow was 0.5 mL min^–1^ and injection volume was 50 μL. Standards, blanks and QAQC samples were analyzed intermittently throughout the run. Triclosan was analyzed in negative electrospray ionization mode with multiple reaction monitoring transitions of 287 to 35 *m/z* and 289 to 37 *m/z* with collision energy of 15 V.

### Culturable Vibrio analysis

To determine culturable *Vibrio* concentrations, replicate bottles (n = 3) from each time point (0 h and 24 h) were mixed vigorously and samples were processed by spread-plating 100 μL onto TCBS (thiosulfate-citrate-bile salts-sucrose) agar in triplicate. Plates were inverted and incubated overnight for 18 h at 30°C after which all yellow and green colonies were counted. Counts were used to determine the number of *Vibrio* colony forming units (CFU) mL^–1^.

### Bacterial DNA Extraction: Looe Key Reef

For the Looe Key Reef microcosms, an additional 900 mL from each microcosm vessel at time zero and time 24 h was filtered onto high volume 0.2 μm Sterivex (Millipore) cartridges, dried, and stored at –20°C for ~1 week before final storage at –80°C. DNA extraction was completed within the Sterivex cartridges after thawing to room temperature. For each cartridge, 1,600 μL of filtered (0.2 μm) lysis buffer (40 mM EDTA, 50 mM Tris (pH 8.3), and 0.73 M sucrose) with lysozyme (2.5 mg mL^–1^) was added though the female Luer-Lock end via pipette, and held for 30 min at 37°C while rotating (7 rpm) in a hybridization oven (Boekel Big SHOT 230400). Following incubation, 100 μL of lysis buffer with Proteinase K (final concentration of 0.75 mg mL^–1^) and 200 μL 10% SDS (final concentration of 1%) were added into the cartridge (total volume of 2 mL). The cartridge was incubated for 2 h at 55°C while rotating in a hybridization oven. Each cartridge was vortexed for a full 30 sec to release DNA. Lysate was transferred into a 5-mL tube where an equal volume of pheno l:chloroform:isoamylalcohol (25:24:1; pH 8.0) was added to the lysate and mixed by inverting the tube until the phases were well mixed. Tubes were centrifuged for 5 min at 3,500 × g at 4°C. The upper aqueous layer was transferred to a new clean 5-mL tube for salt precipitation with 5 M NaCl (0.04 × volume transferred) and isopropanol (0.7 × the volume transferred). These tubes were mixed and incubated at room temperature for 10 min before centrifuging for 15 min at –5°C at 17,000 × g. Supernatant fluid was discarded and the pellet re-suspended in 200 μL of elution buffer (E.Z.N.A. Water DNA kit, Omega BioTek) via vortexing and incubation for 10 min at 65°C. Extracts were stored immediately at –20°C.

### Vibrio and Total Bacterial Quantitative PCR (qPCR): Looe Key Reef

Samples from Looe Key Reef microcosms were analyzed for total *Vibrio* concentrations and were compared to total bacterial levels using SYBR green qPCR. Genus-specific primers for *Vibrio* targeted a variable region of the 16 S rRNA gene (rDNA), 567 F, 5’GGCGTAAAGCGCATGCAGGT-3’ and 680 R, 5’-GAAATTCTACCCCCCTCTACAG-3’ (Thompson et al., 2004). Primers specific for the domain Bacteria, amplified positions 567–680 and 965–1,063 (V6 hypervariable region) of the *E. coli* numbering of the 16 S rRNA gene, 967 F, 5’-CAACGCG AAGAACCTTACC-3’ and 1046 R, 5’-CGACAGCCATGC ANCACCT-3’ (Sogin et al., 2006; Vezzulli et al., 2016). Amplification reactions contained 10 μL of Power Up SYBR Green Master Mix (Applied Biosystems, Foster City, GA), 0.2 μM of each forward and reverse primers, 5 μL of DNA template with molecular grade water for a final volume of 20 μL. All reactions were run in triplicate on a StepOne real-time PCR system (Life Technologies, Grand Isle, NY) under the following cycling conditions: 2 min at 50°C for UDG activation and 95°C for 2 min to activate AmpliTaq polymerase and UP, followed by 40 cycles of 95°C for 15 sec (denaturation) and 60°C for 1 min (annealing and extension) for the *Vibrio*-specific qPCR. The reaction for total bacteria used the same cycling conditions except that the annealing and elongation were split into two steps: 61°C for 15 sec (annealing) and 72°C for 1 min (elongation). Each run was followed by a dissociation step (95°C for 30 sec and 60°C for 30 sec and 95°C for 30 sec) to determine a melt curve for analysis of specificity. Three replicate negative (no template) controls were also included. Cycle threshold (*C*_T_) values were compared to standard curves (equivalent to 10^1^–10^6^ and 10^0^–10^7^ gene copies per reaction volume for the *Vibrio-*specific and total bacterial assays, respectively (See Supplemental Material)). Cell concentrations were expressed as number of cell equivalents (CE) by dividing the sample copy number by 9 (the average 16 S rDNA copy number in *Vibrio*) and 3.5 (the average 16 S rDNA copy number for proteobacteria) (Acinas et al., 2004; Kormas, 2011; Vezzulli et al., 2012).

### Bacterial Community Analysis: Looe Key Reef

Extracted DNA was quantified (NanoDrop 1000, Thermo Scientific, Wilmington, DE) and diluted 1:10 before being subjected to PCR amplification of the 16 S rDNA V4 hypervariable region (primers 515F/806R; Caporaso et al., 2011). Primers contained unique multiplex adaptor barcodes for Illumina sequencing (Tinker and Ottesen, 2016) (Table S2). All samples were run in technical duplicate reactions to account for PCR variability. Two rounds of amplification were used to amplify and tag the V4 16 S rDNA region. The first round of amplification reactions contained 1 × Q5 Buffer (New England BioLabs [NEB], Ipswich, MA), 0.5 μM of each forward and reverse primers, 0.2 mM dNTPs mix (NEB), 0.01 × Q5 Hot Start High-Fidelity DNA polymerase (NEB), and 2 μL of DNA template with molecular grade water for a final volume of 10 μL per reaction. PCR conditions were 98°C for 30 sec for initial denaturation followed by 25 cycles of 10 sec at 98°C, 30 sec at 52°C, 20 sec at 72°C. The final extension step was 72°C for 2 min.

Custom Illumina adaptors with barcode sequences were added during a second round of amplification (Table S2). The second reaction contained 1 × Q5 Buffer (NEB), 0.5 μM of each forward and reverse custom Illumina Barcode Primers, 0.2 mM dNTPs mix (NEB), and 0.01 × Q5 Hot Start High-Fidelity DNA polymerase (NEB) with 9 μL of PCR product from reaction 1 and molecular grade water for a final volume of 30 μL per reaction. PCR conditions were 30 sec at 98°C for initial denaturation followed by 4 cycles of 10 sec at 98°C, 30 sec at 52°C, 20 sec at 72°C for 20 sec, followed by 6 cycles of 10 sec at 98°C, 1 min at 72°C. The final extension step was 72°C for 2 min. Amplicons were purified using equal volume SPRI magnetic beads (Sera-Mag SpeedBeads, Thermo Scientific, Freemont, CA) (Rohland and Reich, 2012) with 96 well magnetic plate (Promega MagnaBot II) and stored at –20°C. Samples were sent to the Georgia Genomics Facility (GGF) (Athens, GA) where they were pooled, tested for quality, and normalized using a fragment analyzer and qPCR. Pooled samples were sequenced using v2 chemistry on an Illumina MiSeq PE250.

### Bioinformatics

Quantitative Insights into Microbial Ecology (QIIME) version 1.9.1 (Caporaso et al., 2010b) was used to merge pairends of Illumina MiSeq reads with *fastq-join*. Merged reads were then imported into Geneious version 8.1.8 (http://www.geneious.com; Kearse et al., 2012) where individually tagged technical replicate PCR files were combined, primers annotated, and trimmed. UCHIME was used to remove chimeric sequences (Edgar et al., 2011) referenced against the RDP Gold reference database. The resulting sequences were used to pick operational taxonomic units (OTUs) with the QIIME pipeline (Wang et al., 2007; Caporaso et al., 2010a, 2010b, Edgar et al., 2010) using open reference and taxonomy assigned with green genes database (gg_13_8. fasta) (DeSantis et al., 2006; McDonald et al., 2012). OTUs were aligned with PyNast and FastTree was used to generate a phylogenetic tree (Price et al., 2010). QIIME was then used to filter unwanted sequences including: mitochondria, archaea, chloroplasts, and singletons. Sequence reads from samples were normalized to the smallest number of reads (179,290 reads) in order to generate data sets with equal abundance. Sampling reads ranged from 179,290 to 1,190,540 with a median of 443,391 reads before subsampling. Sequences were deposited into the NCBI BioProject database (accession no. PRJNA376004).

### Statistical analyses

To determine proportional growth of culturable *Vibrio* under the different microcosms treatments, concentrations at 24 h (T_24_) were divided by the mean concentrations of *Vibrio* at time zero (T_0_). These were averaged (n = 3) to determine the mean proportional growth of *Vibrio* in each microcosm condition (T24/T0). Population growth was noted when T24/T0 was >1 versus population decline when T_24_/T_0_ was <1. All a nalyses were run in GraphPad Prism 7.0 for Mac OS X (GraphPad Software, La Jolla California, USA, www.graphpad.com).

Changes in mean relative *Vibrio* abundance were determined by calculating the *Vibrio* abundance indices (VAI), dividing the *Vibrio* CE ml^–1^ by the total bacterial CE ml^–1^ (Vezzulli et al., 2012, 2016). Changes in VAI were analyzed with one-way ANOVA, after arcsine square root transformation, to determine the main effect of triclosan treatments. If results were found to be significant (α = 0.05), Tukey multiple comparison procedures were followed to determine the differences between treatments.

Bacterial community analyses for Looe Key Reef were conducted in QIIME, GraphPad Prism 7.00 for Mac OS X, and R (R Development Core Team, 2013). Core diversity analyses were run in QIIME to determine alpha and beta diversity parameters. Alpha diversity, Chao1, was calculated to determine within sample diversity. Weighted UniFrac distance matrix (Lozupone and Knight, 2005) was calculated to create principle coordinates analysis (PCoA). PCoA plots were constructed using PhyloToAST 1.3.0 (Dabdoub et al., 2016). Differences in bacterial community composition between samples were determined in QIIME, using the vegan package (Oksanen et al., 2016) in R with permutation-based multivariate analysis (PERMANOVA) using the adonis function, which used the weighted UniFrac metric distance matrix stratified by triclosan treatment.

The relative abundances (proportion) of bacteria at the Family level were arcsine square root-transformed to approximate a normal distribution, and the transformed data were analyzed in ANOVA to determine main effects of treatment on changes in relative composition. If changes in proportions for a Family were found to be significant (α = 0.05), Tukey multiple comparison procedures were followed to determine the differences between treatments.

## Results

### Triclosan concentrations

Background concentrations of triclosan were 103 ng L^–1^ and 362 ng L^–1^ for Looe Key Reef and Doctors Arm Canal, respectively (Table 1). At Clam Bank Landing, triclosan concentrations were 18.4 ng L^–1^, near the limit of detection. The final measured triclosan concentrations for the low triclosan treatments were well below the targeted addition of 5 μg L^–1^ for each experiment (692, 743, and 863 ng L^–1^, for Looe Key Reef, Doctors Arm Canal, and Clam Bank Landing, respectively). Two-tailed t-tests indicated that final concentrations in low triclosan treatments were not significantly different than background levels for all sites (t = 1.645, df = 16, p = 0.1196). The final measured triclosan concentrations for the high triclosan treatments were 4,328, 5,237, and 4,887 μg L^–1^ for Looe Key Reef, Doctors Arm Canal, and Clam Bank Landing, respectively. These values were statistically similar to the targeted 5,000 μg L^–1^ addition.

### Vibrio Proportional Growth

**Figure 3:**
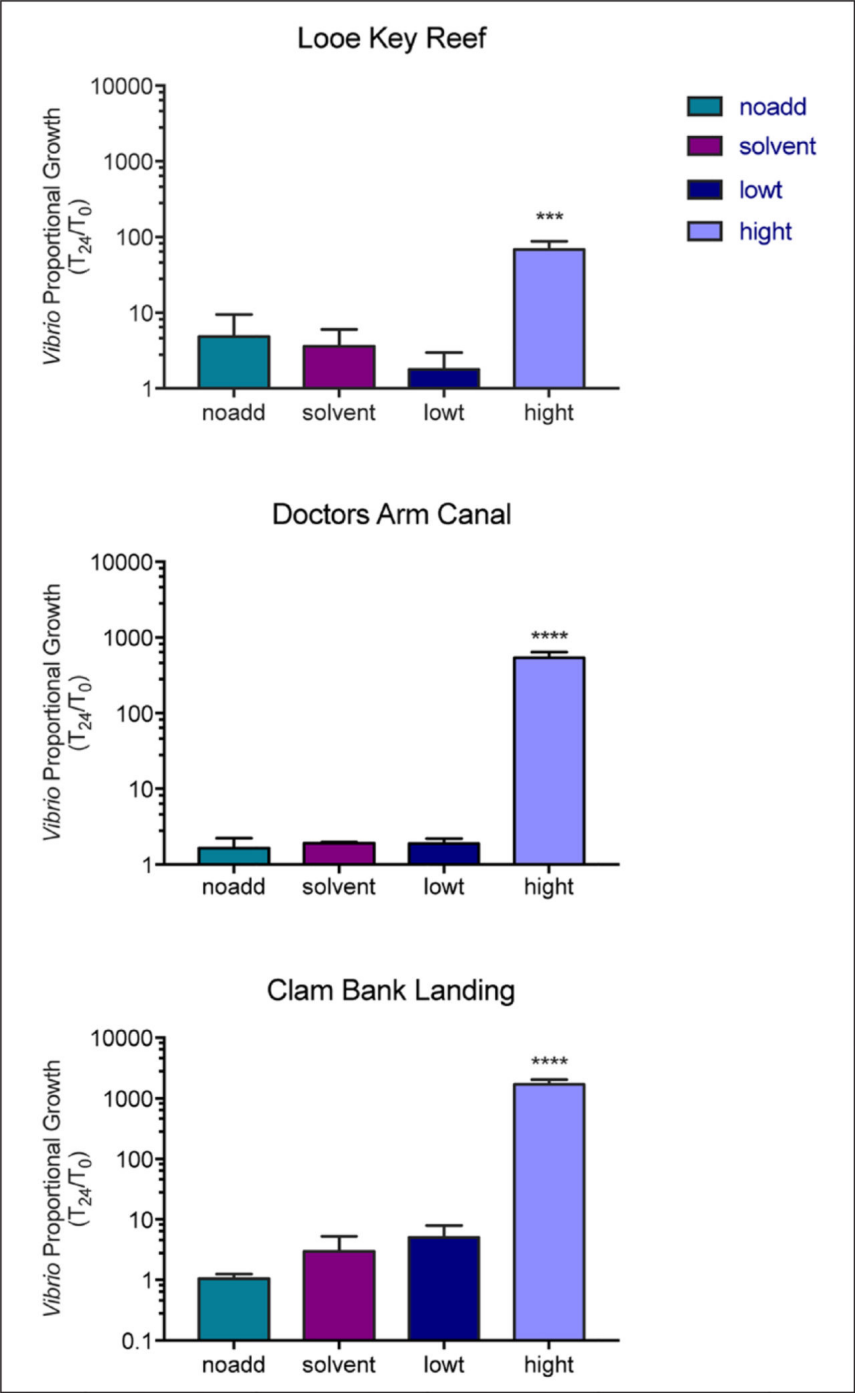
*Vibrio* growth after 24 hours of exposure to triclosan. *Vibrio* concentrations were normalized to time zero to determine the relative growth after 24 h (T_24_/T_0_). Error bars represent SEM (n = 3). Treatments included no triclosan added (noadd), 0.05% ethanol (solvent), low triclosan (lowt), and high triclosan (hight). Locations were Looe Key Reef, offshore Florida Keys, and Doctors Arm Canal, saltwater canal in the Florida Keys (Figure 2), and Clam Bank Landing, North Inlet Estuary, Georgetown, SC (Figure 1). DOI: https://doi.org/10.1525/elementa.141.f3

Mean background levels of *Vibrio* measured at time zero were 64, 134, and 2,000 CFU mL^–1^ for Looe Key Reef, Clam Bank Landing, and Doctors Arm Canal, respectively. For the no addition control, solvent control, and low triclosan treatments (with triclosan levels statistically similar to the no addition levels), *Vibrio* growth at 24 h remained near the levels measured at T_0_, with less than 5-fold change for all experiments (p > 0.05 for all; Figure 3). Conversely, for all sites, high triclosan treatments resulted in significantly higher proportional *Vibrio* growth in comparison with T_0_ (Doctors Arm and Clam Bank, p ≤ 0.0001; Looe Key, p = 0.0002; Figure 3). At 24 h *Vibrio* growth was 68-, 540-, and 1,701-fold higher than T_0_ in high triclosan microcosms for Looe Key Reef, Doctors Arm Canal, and Clam Bank Landing, respectively (Tables S3.1–S3.3). The effect of high triclosan treatments on proportional *Vibrio* growth also differed significantly by site (Looe Key Reef (F_3,8_ = 34.38, p < 0.0001), Doctors Arm Canal (F_3,8_ = 82.45, p < 0.0001) and Clam Bank Landing (F_3,8_ = 77.55, p < 0.0001); Figure 3).

**Figure 4:**
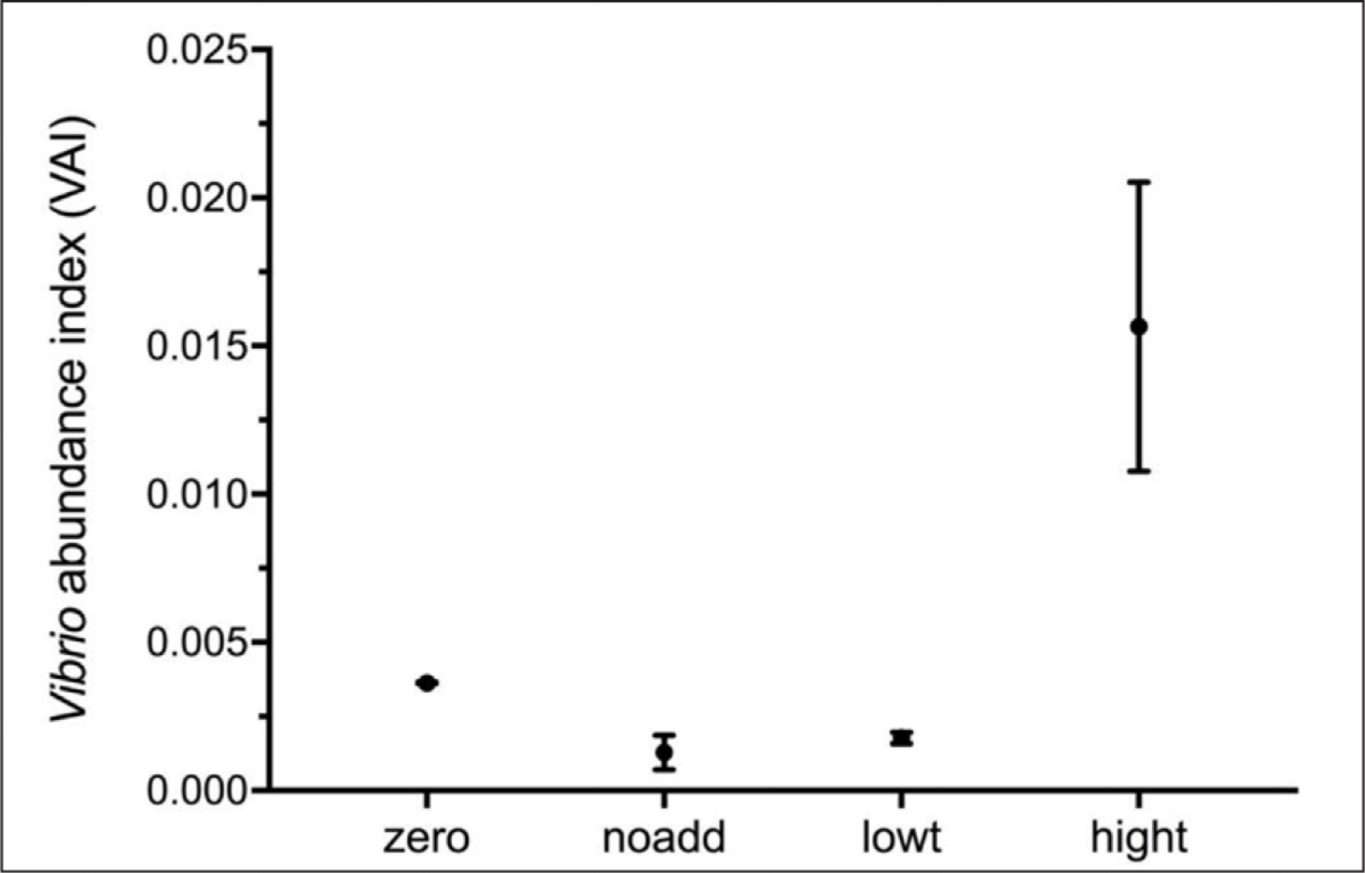
*Vibrio* abundance index (VAI) before and after experimental treatments. VAI was calculated as *Vibrio* CE ml^–1^ divided by the total bacterial CE ml^–1^) at time zero (zero) and after 24 h treatment. Treatments included no triclosan added (noadd), low triclosan (lowt), and high triclosan (hight). Error bars indicate SEM (n = 3). The VAI for high triclosan was significantly greater than all other treatments and the value at time zero (p < 0.01). DOI: https://doi.org/10.1525/elementa.141.f4

The main effects of triclosan treatment were also significant for relative *Vibrio* abundance (VAI as determined through qPCR assays; Table S4) in the Looe Key Reef microcosms in comparison with total bacterial abundance (F_3,8_ = 11.42, p = 0.0029; Figure 4). After 24 h, *Vibrio* made up 0.13% of the total bacterial population in the no addition control and 0.18% in the low triclosan treatment. These values were not significantly different from the 0.36% of the population observed at T_0_. Conversely, *Vibrio* comprised 1.6% of the total bacterial population after 24 h in the high triclosan treatment (Figure 4).

### Bacterial Community Composition: Looe Key Reef

**Figure 5:**
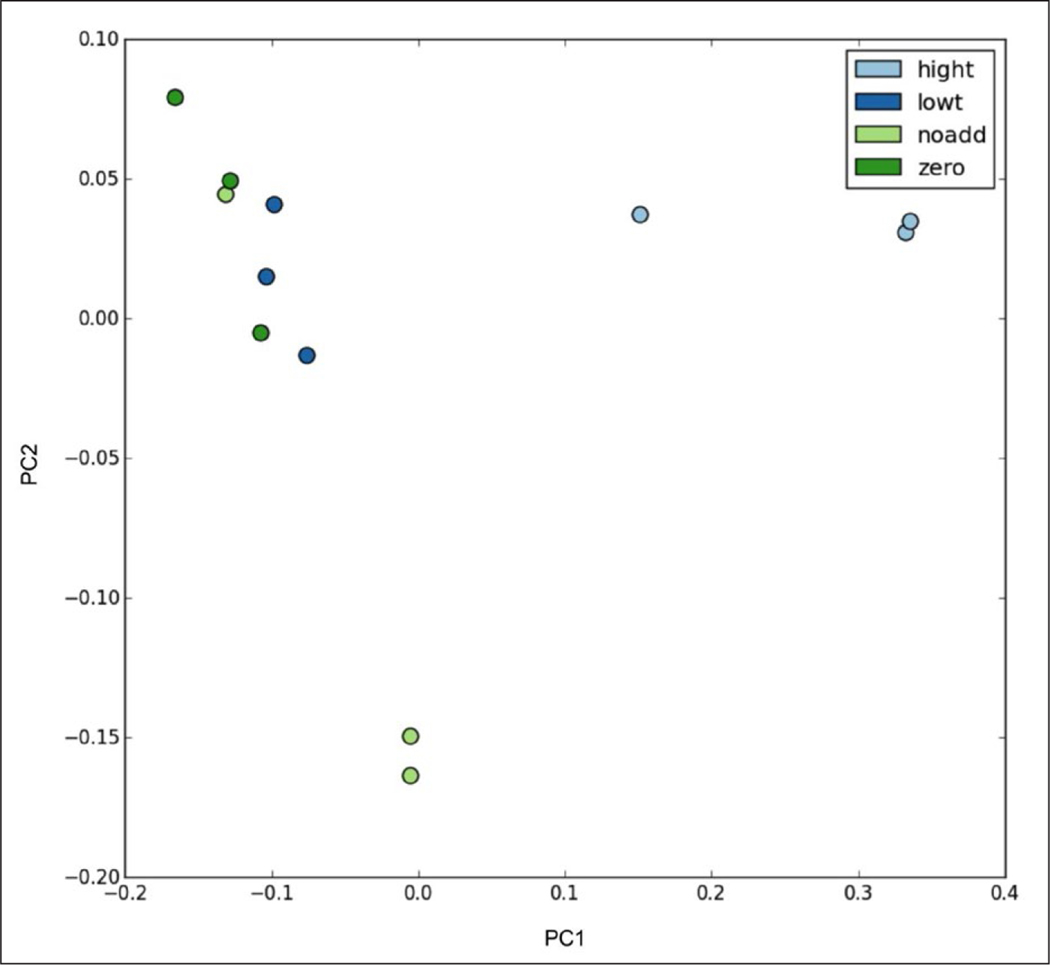
Principle coordinates analysis for triclosan exposure experiments using Looe Key reef surface water. Analysis is based on weighted UniFrac distance metrics. Data points are color-coded for time zero and three treatments at 24 h: no triclosan added (noadd), low triclosan (lowt), and high triclosan (hight). The first principal component (PC1) explained 67.00% of the variation; the second (PC2) explained 12.69%. DOI: https://doi.org/10.1525/elementa.141.f5

The number of observed OTUs among the Looe Key Reef microcosms ranged from 2,793 to 13,213 with an average of 7,165 OTUs. Average (n = 3) Shannon diversity index remained similar across all treatments after 24 h (range 6.72–7.67 H’) and in comparison with T_0_ (7.35), indicating evenness amongst all samples. Additionally, there was little change in richness (Chao1) across the treatments, except for the no addition microcosms after 24 h, which had lower richness in comparison with all others (Table 2). Phylogenetic similarity (weighted UniFrac distance matrix) for bacterial communities in the experimental microcosms for Looe Key reef showed significant differences (p = 0.001; Table S5) with 74.9% of variation in samples attributed to treatment. PCoA showed clustering of high triclosan treatments separately from all other treatments (Figure 5).

To examine bacterial groups impacted by triclosan treatments, bacterial Families were chosen for relative abundance analysis if at least 1% of the total community for a single sample included that Family and the Family group was assigned a definitive Family name. In total, 18 bacterial Families were selected using these criteria (out of 490) and compared for effects of triclosan treatment on relative abundance.

For the low triclosan additions, only one family showed significant changes in post hoc analyses for relative abundance after 24 h exposure. The relative abundance of OM60 increased by 4.5 fold after 24 h (p = 0.0002); however, OM60 also showed growth in the no addition controls (increased by 2.9 fold), indicating that bottle effects may have been important, which is consistent with the observation that triclosan concentration in the low triclosan treatment did not differ from the no addition control. All other bacterial Family groups showed no change at 24 h after exposure to low triclosan.

**Figure 6:**
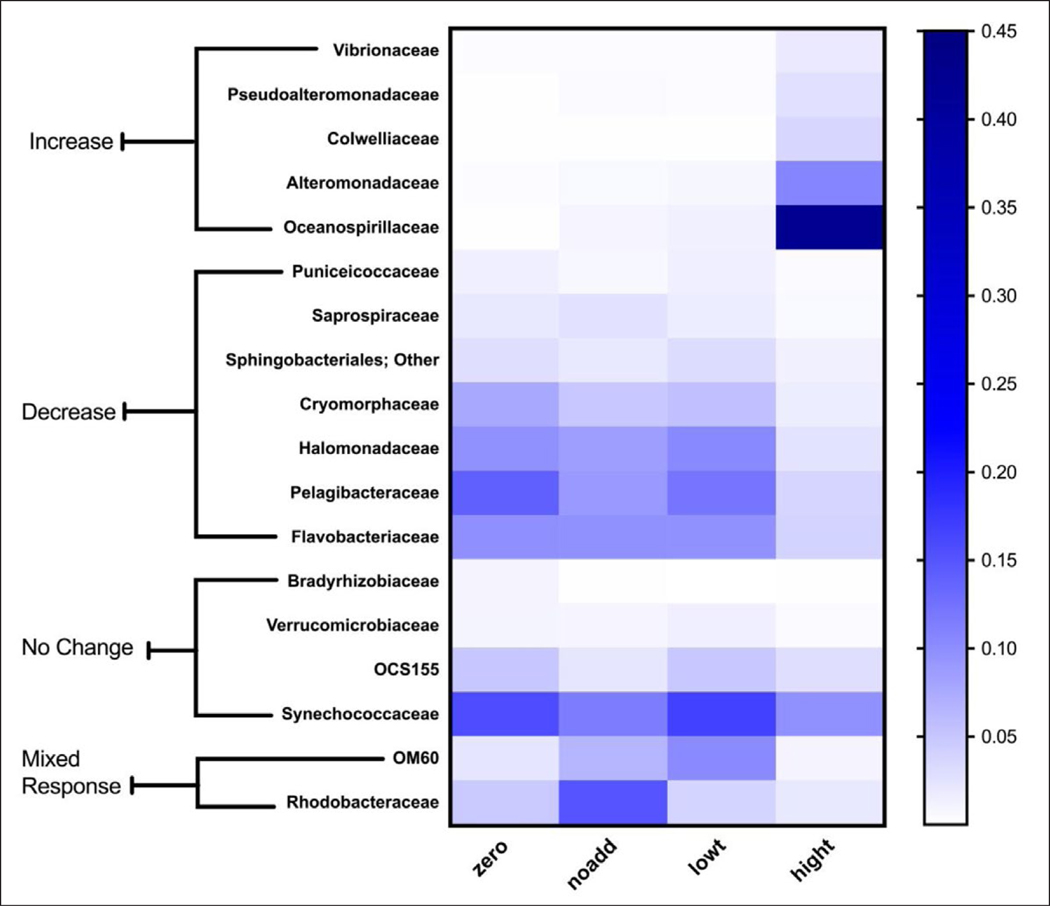
Relative abundance of 18 bacterial Families in natural seawaters (Looe Key Reef) exposed to triclosan. Color scale indicates bacterial proportional abundance for time zero and three treatments at 24 h (n = 3): no triclosan added (noadd), 0.05% ethanol (solvent), low triclosan (lowt), and high triclosan (hight). Families included in the Increase and Decrease categories demonstrated shifts in their relative abundances after 24-h exposure to high concentrations of triclosan that differed significantly from that observed at time zero (p < 0.01). For families categorized as No Change, there was no statistically significant difference in relative abundance between the treatments and control. The group labeled as Mixed Response, included families that showed significant but inconsistent shifts in relative abundance compared to controls, likely due to bottle effects. DOI: https://doi.org/10.1525/elementa.141.f6

At 24-h exposure under the high triclosan treatment, 12 Families demonstrated significant changes in relative abundance compared to time zero. High triclosan treatments resulted in significantly greater relative abundance for five families: Vibrionaceae (17-fold), Pseudoalteromonadaceae (65-fold), Alteromonadaceae (108-fold), Colwelliaceae (430-fold), and Oceanospirillaceae (1494-fold) (Figure 6). Conversely, Cryomorphaceae, Flavobacteriaceae, Halomonadaceae, Pelagibacteraceae, Puniceicoccaceae, Saprospiraceae, and Sphingobacteriales- Other decreased by 60–80% in relative abundance for high triclosan treatments in comparison with time zero (Figure 6). Two families showed mixed responses to both no addition and low triclosan controls (OM60 [p = 0.0046] Puniceicoccaceae, Saprospiraceae, and SphingobacterialesOther decreased by 60–80% in relative abundance for high triclosan treatments in comparison with time zero (Figure 6). Two families showed mixed responses to both no addition and low triclosan controls (OM60 [p = 0.0046] and Rhodobacteraceae [p = 0.0010]) and an additional four families showed no significant changes with any condition (Figure 6; Table S6).

## Discussion

Antimicrobials make up a large portion of PPCPs entering the environment from treated wastewater effluent (Kolpin et al., 2002; Ellis, 2006; Hedgespeth et al., 2012; Guerra et al., 2014). Properly working secondary treatment plants can remove up to 95% of the incoming triclosan using activated sludge, but even at 95% removal, significant concentrations remain in both biosolids and in final discharge (McAvoy et al., 2002; Thompson et al., 2005). Removal through other disposal systems, such as septic systems which are common in coastal communities, are even less effective (Matamoros et al., 2009; Yang et al., 2016). A ban on triclosan in hand soaps by the U.S. Food and Drug Administration on September 9, 2016 (U.S. Food and Drug Administration, HHS 2016) will phase out triclosan use in hand soaps; however, triclosan will have continued use in toothpastes and other products and is expected to persist in sediments for years (Singer et al., 2002; Miller et al., 2008). Given such a potentially large impact, it is important to understand the role these antimicrobials may play in the ecology of coastal aquatic bacteria and potential bacterial pathogens.

In this study, background levels of triclosan in coastal surface waters for Looe Key Reef and Clam Bank Landing were similar to previously reported concentrations in seawater (≤100 ng L^–1^) (Okumura and Nishikawa, 1996; Wu et al., 2007; Xie et al., 2008; Fair et al., 2009); however, concentration from surface water from Doctors Arm Canal (362 ng L^–1^) was over three times greater than prior reports in coastal areas. Background levels of triclosan may already be providing some selective pressure in the environment. Unfortunately, we were not able to see laboratory effects from a modest increase in triclosan concentration because the additions were far lower than expected, with final concentrations no different from starting values. This issue likely contributed to the lack of *Vibrio* response and minimal observed effects on microbial communities in the low triclosan treatment. However, previous research has shown that triclosan can induce antibiotic resistance in the environment at levels as low as 500 ng L^–1^ (Nietch et al., 2013; Carey and McNamara, 2015). This induction of resistance indicates that while the community may not change nor have increased abundance of pathogens with low doses of triclosan, there could be additional risks in terms of cross- or co-resistance to important clinical antibiotics that are used to treat infections but were not addressed in our study design.

Although our focus was on surface waters, the high triclosan concentrations tested were in range for what has previously been reported in sediments (<100–53,000 μg kg^–1^ dry weight; Okumura and Nishikawa, 1996; Singer et al., 2002; Morales et al., 2005; Miller et al., 2008; SCCS, 2010). Results in this study demonstrated that exposure to the PPCP triclosan at high doses of ~5,000 μg L^–1^ (~5 ppm) has a significant effect on both *Vibrio* growth dynamics and marine microbial communities in natural seawater microcosms. These results were comparable with previous research that showed significant growth of *Vibrio* bacteria in grass shrimp homogenate after exposure at a lower 330 ppb triclosan concentration (DeLorenzo et al., 2014). Results here and from the prior study suggest that a *Vibrio* growth response is common in the presence of elevated triclosan concentrations, even within different habitat types. Given that triclosan is known to persist in sediments for long periods of time (Singer et al., 2002; Miller et al., 2008), human interventions, such as dredging, and natural events, such as hurricanes, could disturb deposited triclosan from sediments into the water column and potentially induce blooms of *Vibrio* bacteria. In estuarine systems, influx of marine waters may also allow triclosan to move from the sediment to the dissolved phase in water during incoming tides (Miller et al., 2008). Our results are consistent with earlier studies, which reported *Vibrio* enrichment in surface waters exposed to pharmaceutical wastes (Peele et al., 1981; Grimes et al., 1984).

Under high triclosan conditions (ppm), taxa from five families (Vibrionaceae, Pseudoalteromonadaceae, Alteromonadaceae, Colwelliaceae, and Oceanospirillaceae) significantly expanded their representation in the oligotrophic surface water community of Looe Key reef (Szmant and Forrester, 1996). Furthermore, these families (all members of the class Gammaproteobacteria) were found in very low abundance (<0.1%) in time zero microcosms and could be considered conditionally rare taxa. Conditionally rare taxa are able to impact ecosystem function during bloom conditions; however, little is known about the environmental drivers that can induce blooms (Yooseph et al., 2010; Campbell et al., 2011; Shade et al., 2014). Results from this study suggest that elevated concentrations of triclosan are able to induce bloom conditions for some of these conditionally rare taxa. This finding is significant because it suggests the potential for xenobiotics in addition to triclosan to act as drivers of microbial ecology in coastal ecosystems.

Increases in the relative abundance of key bacterial families in high triclosan microcosms could be due to a number of factors, including: 1) presence of pathways for resistance to triclosan across these groups, 2) an ability to use triclosan as a carbon source, and/or 3) an ability to use dead bacteria from susceptible groups as a carbon source in this oligotrophic system. While the Vibrionaceae abundance increased significantly after 24 h, the increase was relatively modest; however, even a modest expansion is important given the large number of known human and animal pathogens in this group and the confirmed resistance of *V. cholerae* to triclosan (Massengo-Tiassé and Cronan, 2008). Conversely, Oceanospirillaceae expanded by 1,494-fold in high triclosan microcosms. Previous research has shown that some species within this family are known for their abilities to degrade oil and thrive in hydrocarbon rich environments (Yakimov et al., 2003; Teramoto et al., 2009; Smith et al., 2015). It is possible that members of the Oceanospirillaceae have unique adaptations related to oil degradation (Yakimov et al., 2003; Teramoto et al., 2009) or dissolved inorganic carbon assimilation (DeLorenzo et al., 2012) that make them able to utilize and/or avoid the antimicrobial properties of triclosan. Previous research has indicated that a few bacteria, *Pseudomonas putida, Alcaligenes xylosoxidans*, and *Sphingomonas*-like organism strain Rd1, from soils and activated sludge are able to metabolize triclosan as a carbon source (Hay et al., 2001; Meade et al., 2001). To date, no studies have specifically addressed this capacity among aquatic b acteria (neither freshwater nor marine). However, previous research in marine waters showed that heterotrophic orders Alteromonadales (including Alteromonadaceae, Colwelliaceae, Pseudoalteromonadaceae) and Oceanospirillales (including Oceanospirillaceae) contribute significantly to assimilation of dissolved inorganic carbon among Gammaproteobacteria (DeLorenzo et al., 2012). Additionally, genera in the Oceanospiralles family (e.g., *Alcinovorax*) are able to degrade halogenated compounds, supporting the notion that members of this group may be capable of metabolizing triclosan (Li and Shao, 2014). It should also be noted that earlier changes to the community might occur with exposure to triclosan. Effects noted here, after 24 h, may mask very fast initial responses that could inform the mechanism of response.

Pseudoalteromonadaceae, Alteromonadaceae, Colwelliaceae, and Oceanospirillaceae have not previously been reported to be resistant to triclosan. Resistance to triclosan has been noted in some bacteria including the pathogens *Pseudomonas aeruginosa, V. cholerae, E. coli*, and *Salmonella enterica* through numerous pathways including efflux pumps and alternative or mutated enzymes in place of the target enzyme, FabI (McMurry et al., 1998; Chuanchuen et al., 2003; Braoudaki and Hilton, 2004; Gomez Escalada et al., 2005; Yazdankhah et al., 2006; Massengo-Tiassé and Cronan, 2008). Among these known triclosan-resistant bacteria, only members of *Vibrio* (Vibrionaceae family) were observed to increase under triclosan pressure in this study. Moreover, Vibrionaceae is the only bacterial family we observed to increase that also includes pathogenic taxa, which is an important consideration given the notable increase in reported *Vibrio* illnesses in the last 20 years (Newton et al., 2012; U.S. Centers for Disease Control, 2016).

Results from this study suggest that triclosan has the potential to promote *Vibrio* growth in marine and estuarine environments; however, this effect is highly dependent on specific triclosan concentrations. Triclosan accumulates to higher levels in the sediment compared to the overlying water column (Miller et al., 2008; SCCS, 2010) where it could likely impact sediment-associated community where *Vibrio* concentrations exceed levels in the water column by ~2 orders of magnitude (Givens et al., 2014). Furthermore, risks of selection for *Vibrio* could also be enhanced in bivalve shellfish, which are a common exposure route for *Vibrio* infections (Slayton et al., 2014; Froelich et al., 2016). Bioaccumulation of triclosan in clams and mussels has been noted at concentrations ranging from 1.7 to 2578 μg kg^–1^ (Gatidou et al., 2010; Kookana et al., 2013; Álvarez-Muñoz et al., 2015).

## Conclusions

Coastal ecosystems are continuously impacted by PPCPs; however, little is known about the effects these compounds may have on microbial ecology and pathogen population dynamics. This knowledge gap pertains especially to the marine bacteria *Vibrio*, which have been shown to be resistant to the PPCP triclosan (Massengo-Tiassé and Cronan, 2008; DeLorenzo et al., 2014). Findings reported here are essential building blocks to begin to understand the role antimicrobials play in influencing bacterial community structure for wastewater impacted coastal ecosystems. Results presented in this study demonstrated that triclosan selects for *Vibrio* bacteria, and their parent Family Vibrionaceae, in natural seawater communities at triclosan concentrations similar to that reported in sediments (Okumura and Nishikawa, 1996; Singer et al., 2002; Morales et al., 2005; Miller et al., 2008; SCCS, 2010). An enrichment effect was observed for culturable *Vibrio* growth with 64- to 1701-fold increases after 24 h of high triclosan exposure for all stations, representing very different ecosystems and anthropogenic impacts. Based on data presented here and elsewhere, we expect the threshold level of triclosan that promotes *Vibrio* growth falls in the parts per billion range (DeLorenzo et al., 2014). This study, to our knowledge, is unique in demonstrating shifts in microbial communities and selection of *Vibrio* bacteria when exposed to triclosan in natural seawater. Moreover, observed shifts in bacterial community composition with high triclosan exposures suggest new subgroups of aquatic bacteria that could be resistant to triclosan and/or have the ability to utilize triclosan as a carbon source. Results from this study also indicate antimicrobial PPCPs are able to induce bloom conditions of conditionally rare taxa, thus suggesting that xenobiotics could be unrecognized drivers of microbial ecology in coastal ecosystems.

## Supplementary Material

Figure S1 **Figure S1.** Top-Down Effects of Grazer Removal. DOI: https://doi.org/10.1525/elementa.141.s10

Table S6 **Table S6.** ANOVA statistics for changes in relative abundance of 18 bacterial families in Looe Key Reef triclosan microcosms. DOI: https://doi.org/10.1525/elementa.141.s9

Table S5 **Table S5.** Permanova table for the analysis of the weighted UniFrac distance matrix to test the main effects of triclosan treatment on natural seawater bacterial communities. DOI: https://doi.org/10.1525/elementa.141.s8

Table S3.2 **Table S3.2.** Doctors Arm Canal: *Vibrio* spp. concentrations in natural seawater microcosms. DOI: https://doi.org/10.1525/elementa.141.s5

Table S4 **Table S4.** qPCR cell equivalents per mL for calculation of *Vibrio* abundance index (VAI) (*Vibrio* CE mL–1/total bacterial CE mL–1) for Looe Key experiment. DOI: https://doi.org/10.1525/elementa.141.s7

Table S3.3 **Table S3.3.** Clam Bank Landing: *Vibrio* spp. concentrations in natural seawater microcosms. DOI: https://doi.org/10.1525/elementa.141.s6

Table S2 **Table S2.** Barcodes for forward and reverse primers used for 16 S rDNA sample tagging. DOI: https://doi.org/10.1525/elementa.141.s3

Table S1 **Table S1.** Mean (and std error, SEM) Triclosan Concentrations for Glass vs. Plastic. DOI: https://doi.org/10.1525/elementa.141.s2

Table S3.1 **Table S3.1.** Looe Key Reef: *Vibrio* spp. concentrations in natural seawater microcosms. DOI: https://doi.org/10.1525/elementa.141.s4

Supplementary Data 3

Supplemental Material **Text S1.** Supplemental Material. DOI: https://doi.org/10.1525/elementa.141.s1

Supplementary Data 2

Supplementary Data 1

## Figures and Tables

**Table 1:** Average triclosan concentrations measured in the seawater microcosms. DOI: https://doi.org/10.1525/elementa.141.t1

Seawater location	Condition	Triclosan (μg L^−1^)	SEM^d^
Looe Key Reef	T_0_^a^	0.103	0.037
	Low triclosan^b^	0.692	0.173
	High triclosan^c^	4,330	277
Doctors Arm Canal	T_0_	0.362	0.142
	Low triclosan^b^	0.743	0.068
	High triclosan^c^	5,240	489
Clam Bank Landing	T_0_	0.018	0.012
	Low triclosan^b^	0.863	0.144
	High triclosan^c^	4,890	620

a Time zero; i.e., pre-addition level of triclosan in the seawater used in the microcosms.

b Actual amount added for the low triclosan treatment was 13.84, 14.64, and 17.26% of the intended 5,000 ng L^−^1 addition for Looe Key, Doctors Arm, and Clam Bank, respectively.

c Actual amount added for the high triclosan treatment was 86.56, 104.7, and 97.74% of the intended 5,000 μg L^−1^ addition for Looe Key, Doctors Arm, and Clam Bank,respectively.

d Standard error of the mean, n = 3.

**Table 2:** Mean alpha diversity summary metrics for Looe Key Reef microcosm microbial communities. DOI: https://doi.org/10.1525/elementa.141.t2

Condition	Sample size (n)	OTUs (#)	Chao1 (#)^b^	Shannon (H’)^c^
Time zero	3	6507	11347	7.35
No addition^a^	3	6038	9768	7.28
Low triclosan^a^	3	9308	18245	7.67
High triclosan^a^	3	6807	12473	6.71

a At 24 h

b An estimate of richness

c An index of diversity
