# Supplementary figures and images for: Effects of triclosan on bacterial community composition and Vibrio populations in natural seawater microcosms
Source: Elementa (Wash D C). Author manuscript; Available in PMC 2022 Feb 16. (PMC8849560; doi:10.1525/elementa.141)

**Figure S1. Top-Down Effects of Grazer Removal.**

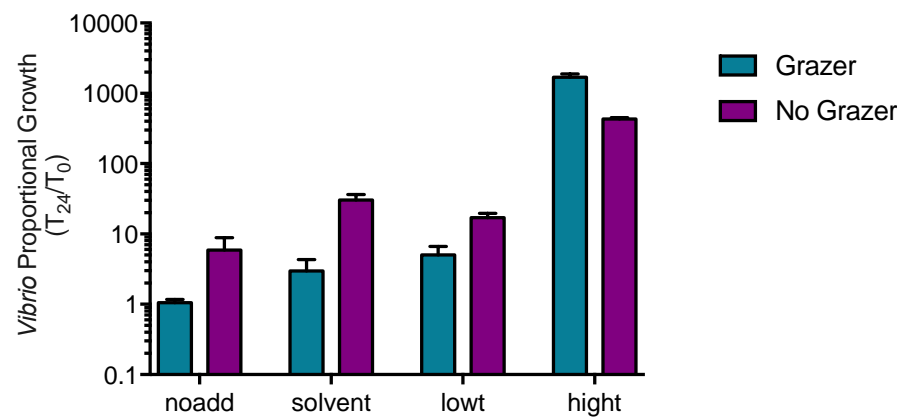

Supplement: Figure S1 — Top-Down Effects of Grazer Removal. DOI: https://doi.org/10.1525/elementa.141.s10 [file NIHMS1048548-supplement-Figure_S1.pdf]
