# Supplementary material for: Effects of triclosan on bacterial community composition and Vibrio populations in natural seawater microcosms
Source: Elementa (Wash D C). Author manuscript; Available in PMC 2022 Feb 16. (PMC8849560; doi:10.1525/elementa.141)
Supplement: Table S6 — ANOVA statistics for changes in relative abundance of 18 bacterial families in Looe Key Reef triclosan microcosms. DOI: https://doi.org/10.1525/elementa.141.s9 [file NIHMS1048548-supplement-Table_S6.pdf]

Table S6. ANOVA statistics for changes in relative abundance of 18 bacterial families in Looe Key Reef triclosan microcosms.

If differences by ANOVA were determined to be statistically significant, Tukey multiple comparisons test was run comparing treatments (noadd, lowt, hight) to relative abundance at time zero (zero).

	ANOVA		Tukey Multiple Comparisons p-values		
**Bacterial Family**	**$F_{3,8}$ =**	**p-value**	**zero v noadd**	**zero v lowt**	**zero v hight**
Alteromonadaceae	58.72	< 0.0001	0.4562	0.2779	<0.0001
Bradyrhizobiaceae	3.801	0.0581	-	-	-
Colwelliaceae	35.22	< 0.0001	0.9999	0.9751	0.0001
Cryomorphaceae	27.36	0.0001	0.1178	0.3202	0.0001
Flavobacteriaceae	33.47	< 0.0001	0.9992	0.8447	0.0001
Halomonadaceae	10.44	0.0039	0.9972	0.8046	0.0122
Oceanospirillaceae	59.8	< 0.0001	0.6576	0.6466	< 0.0001
OCS155	2.433	0.14	-	-	-
OM60	43.35	< 0.0001	0.0046	0.0002	0.1545
Pelagibacteraceae	7.45	0.0105	0.5474	0.8998	0.0101
Pseudoalteromonadaceae	82.12	< 0.0001	0.1177	0.4241	< 0.0001
Puniceicoccaceae	12.47	0.0022	0.2636	0.7945	0.0066
Rhodobacteraceae	30.95	< 0.0001	0.001	0.9852	0.0849
Saprospiraceae	8.731	0.0066	0.3409	>0.9999	0.0485
Sphingobacteriales; other	14.26	0.0014	0.3269	0.8177	0.0038
Synechococcaceae	1.435	0.3028	-	-	-
Verrucomicrobiaceae	5.544	0.0235	0.9965	0.5763	0.1058
Vibrionaceae	17.65	0.0007	0.9925	0.9783	0.002
