# Supplementary material for: Effects of triclosan on bacterial community composition and Vibrio populations in natural seawater microcosms
Source: Elementa (Wash D C). Author manuscript; Available in PMC 2022 Feb 16. (PMC8849560; doi:10.1525/elementa.141)
Supplement: Table S5 — Permanova table for the analysis of the weighted UniFrac distance matrix to test the main effects of triclosan treatment on natural seawater bacterial communities. DOI: https://doi.org/10.1525/elementa.141.s8 [file NIHMS1048548-supplement-Table_S5.pdf]

**Table S5. Permanova table for the analysis of the weighted UniFrac distance matrix to test the main effects of triclosan treatment on natural seawater bacterial communities.**

compare_categories.py --method adonis -i weighted_unifrac_dm.txt -m MappingFileR.txt -c Treatment -o adonisTri_out -n 999

| | Df | SumsOfSqs | MeanSqs | F.Model | R2 | Pr(>F) |
|---|---|---|---|---|---|---|
| Triclosan treatment | 3 | 0.3838 | 0.1279 | 7.9603 | 0.7491 | 0.001 *** |
| Residuals | 8 | 0.1286 | 0.0161 | | 0.2509 | |
| Total | 11 | 0.5123 | | | 1.0000 | |

Signif. Codes: 0 ‘***’ 0.001 ‘**’ 0.01 ‘*’ 0.05 ‘.’ 0.1 ‘ ‘ 1
