# Supplementary material for: Effects of triclosan on bacterial community composition and Vibrio populations in natural seawater microcosms
Source: Elementa (Wash D C). Author manuscript; Available in PMC 2022 Feb 16. (PMC8849560; doi:10.1525/elementa.141)
Supplement: Table S3.2 — Doctors Arm Canal: Vibrio spp. concentrations in natural seawater microcosms. DOI: https://doi.org/10.1525/elementa.141.s5 [file NIHMS1048548-supplement-Table_S3_2.pdf]

Table S3.2. Doctors Arm Canal: *Vibrio* spp. concentrations in natural seawater microcosms.

T_0 mean CFU mL^{-1} (n = 3) is 2000 CFU mL^{-1}.

**Treatment**	**T_{24} (CFU mL^{-1})**	**T_{24}/mean T_0**	**Mean T_{24}/T_0 (n = 3)**
No addition	3500	1.75	1.65
	2067	1.03	
	4333	2.17	
Solvent control	3667	1.83	1.91
	3867	1.93	
	3933	1.97	
Low triclosan	3400	1.70	1.89
	3400	1.70	
	4533	2.27	
High triclosan	1317333	658.67	540.22
	972000	486.00	
	952000	476.00	
