# Supplementary material for: Effects of triclosan on bacterial community composition and Vibrio populations in natural seawater microcosms
Source: Elementa (Wash D C). Author manuscript; Available in PMC 2022 Feb 16. (PMC8849560; doi:10.1525/elementa.141)
Supplement: Table S4 — qPCR cell equivalents per mL for calculation of Vibrio abundance index (VAI) (Vibrio CE mL–1/total bacterial CE mL–1) for Looe Key experiment. DOI: https://doi.org/10.1525/elementa.141.s7 [file NIHMS1048548-supplement-Table_S4.pdf]

Table S4. qPCR cell equivalents per mL for calculation of *Vibrio* abundance index (VAI) (*Vibrio* CE mL^{-1}/total bacterial CE mL^{-1}) for Looe Key experiment.

Treatment	*Vibrio* CE mL^{-1}	total bacterial CE mL^{-1}	VAI	Mean VAI (n = 3)
Time zero	70.9	1.93×10^4	0.0037	0.0036
	4.4	1.21×10^3	0.0036	
	68.5	1.91×10^4	0.0036	
No addition (24 h)	59.9	4.80×10^4	0.0012	0.0013
	165	7.11×10^4	0.0023	
	12.1	3.94×10^4	0.0003	
Low triclosan (24 h)	71.2	3.35×10^4	0.0021	0.0018
	76.7	4.41×10^4	0.0017	
	50.6	3.43×10^4	0.0015	
High triclosan (24 h)	2.58×10^3	1.44×10^5	0.0179	0.0157
	5.77×10^3	2.54×10^5	0.0228	
	1.87×10^3	2.96×10^5	0.0063	
