# Supplementary material for: Effects of triclosan on bacterial community composition and Vibrio populations in natural seawater microcosms
Source: Elementa (Wash D C). Author manuscript; Available in PMC 2022 Feb 16. (PMC8849560; doi:10.1525/elementa.141)
Supplement: Table S3.3 — Clam Bank Landing: Vibrio spp. concentrations in natural seawater microcosms. DOI: https://doi.org/10.1525/elementa.141.s6 [file NIHMS1048548-supplement-Table_S3_3.pdf]

Table S3.3. Clam Bank Landing: *Vibrio* spp. concentrations in natural seawater microcosms.

T_0 mean CFU mL^{-1} (n = 3) is 134 CFU mL^{-1}.

**Treatment**	**T_{24} (CFU mL^{-1})**	**T_{24}/mean T_0**	**Mean T_{24}/T_0 (n = 3)**
No addition	150	1.12	1.05
	110	0.82	
	163	1.21	
Solvent control	223	1.66	2.98
	757	5.65	
	217	1.62	
Low triclosan	390	2.91	5.06
	537	4.00	
	1107	8.26	
High triclosan	279467	2085.57	1701.50
	198133	1478.60	
	206400	1540.30	
