# Supplementary material for: Effects of triclosan on bacterial community composition and Vibrio populations in natural seawater microcosms
Source: Elementa (Wash D C). Author manuscript; Available in PMC 2022 Feb 16. (PMC8849560; doi:10.1525/elementa.141)
Supplement: Table S2 — Barcodes for forward and reverse primers used for 16 S rDNA sample tagging. DOI: https://doi.org/10.1525/elementa.141.s3 [file NIHMS1048548-supplement-Table_S2.pdf]

Table S2. Barcodes for forward and reverse primers used for 16S rDNA sample tagging.

Sample Name	R-primer barcode	F-primer barcode
LK1_T1A_1	CGTAGCAT	AACCAACC
LK10_HT1_1	CGTAGCAT	CGTTCGTT
LK11_HT2_1	CGTAGCAT	GCAAGCAA
LK12_HT3_1	CGTAGCAT	TTCGTTCG
LK13_T1A_2	TTCGTTCG	AACCAACC
LK14_T1B_2	TTCGTTCG	CCAACCAA
LK15_T1C_2	TTCGTTCG	GGTTGGTT
LK16_NA1_2	CCAACGTA	CCATCCTA
LK17_NA2_2	TTCGTTCG	AGTCGACT
LK18_NA3_2	TTCGTTCG	CCATCCTA
LK19_LT1_2	TTCGTTCG	GTCAAGAG
LK2_T1B_1	CGTAGCAT	CCAACCAA
LK20_LT2_2	CCAACGTA	TAGGTTGC
LK21_LT3_2	TTCGTTCG	AAGCAAGC
LK22_HT1_2	TTCGTTCG	CGTTCGTT
LK23_HT2_2	TTCGTTCG	GCAAGCAA
LK24_HT3_2	TTCGTTCG	TTCGTTCG
LK3_T1C_1	CGTAGCAT	GGTTGGTT
LK4_NA1_1	CCAACGTA	AGTCGACT
LK5_NA2_1	CGTAGCAT	AGTCGACT
LK6_NA3_1	CGTAGCAT	CCATCCTA
LK7_LT1_1	CGTAGCAT	GTCAAGAG
LK8_LT2_1	CCAACGTA	GTCAAGAG
LK9_LT3_1	CGTAGCAT	AAGCAAGC
