# Supplementary material for: Effects of triclosan on bacterial community composition and Vibrio populations in natural seawater microcosms
Source: Elementa (Wash D C). Author manuscript; Available in PMC 2022 Feb 16. (PMC8849560; doi:10.1525/elementa.141)
Supplement: Table S1 — Mean (and std error, SEM) Triclosan Concentrations for Glass vs. Plastic. DOI: https://doi.org/10.1525/elementa.141.s2 [file NIHMS1048548-supplement-Table_S1.pdf]

Table S1. Mean (and std error, SEM) Triclosan Concentrations for Glass vs. Plastic.

	High dose (µg L^{-1})	SEM	Low dose (µg L^{-1})	SEM
Glass to glass	5,918	93	5.399	0.841
Plastic to glass	7,458	692	7.351	0.714
Plastic to plastic	6,440	1,236	6.926	1.143
Glass to Plastic	7,103	947	6.103	1.049
