# Supplementary material for: Effects of triclosan on bacterial community composition and Vibrio populations in natural seawater microcosms
Source: Elementa (Wash D C). Author manuscript; Available in PMC 2022 Feb 16. (PMC8849560; doi:10.1525/elementa.141)
Supplement: Table S3.1 — Looe Key Reef: Vibrio spp. concentrations in natural seawater microcosms. DOI: https://doi.org/10.1525/elementa.141.s4 [file NIHMS1048548-supplement-Table_S3_1.pdf]

Table S3.1. Looe Key Reef: *Vibrio* spp. concentrations in natural seawater microcosms.

T_0 mean CFU mL^{-1} (n = 3) is 64 CFU mL^{-1}.

**Treatment**	**T_{24} (CFU mL^{-1})**	**T_{24}/mean T_0**	**Mean T_{24}/T_0 (n = 3)**
No Addition	67	1.04	4.81
	640	10.00	
	217	3.39	
Solvent Control	90	1.41	3.57
	397	6.20	
	200	3.12	
Low Triclosan	203	3.17	1.79
	67	1.05	
	73	1.14	
High Triclosan	5090	79.53	68.6
	5060	79.06	
	3023	47.23	
