# Supplementary material for: Effects of triclosan on bacterial community composition and Vibrio populations in natural seawater microcosms
Source: Elementa (Wash D C). Author manuscript; Available in PMC 2022 Feb 16. (PMC8849560; doi:10.1525/elementa.141)
Supplement: Supplemental Material — Text S1. Supplemental Material. DOI: https://doi.org/10.1525/elementa.141.s1 [file NIHMS1048548-supplement-Supplemental_Material.pdf]

Effects of triclosan on bacterial community composition and *Vibrio* populations in natural seawater microcosms

Keri Ann Lydon[1], Donna A. Glinski[1,2], Jason R. Westrich[1], W. Matthew Henderson[3], and Erin K. Lipp[1,*]

Department of Environmental Health Science, University of Georgia, Athens, Georgia[1]
Oak Ridge Institute of Science and Education, U.S. Environmental Protection Agency, Athens, Georgia[2]
U.S. Environmental Protection Agency, Office of Research and Development, NERL/EMMD, Athens, Georgia[3]

*Address correspondence to Erin Lipp, elipp@uga.edu

Supplemental Material

Quantitative PCR Standards

Quantitative PCR (qPCR) standards for both the *Vibrio*-specific and total bacteria qPCR were prepared from genomic DNA extracted from *V. alginolyticus* (American Type Culture Collection strain 33839) using a DNeasy Blood and Tissue Kit (Qiagen, Valencia, CA) according to the manufacturer's protocol for Gram-negative bacteria. The extracted genomic DNA was purified using equal volume SPRI magnetic beads (Sera-Mag SpeedBeads, Thermo Scientific, Freemont, CA) (Rohland and Reich, 2012) with 96 well magnetic plate (Promega MagnaBot II) quantified using a Qubit Fluorometer (Thermo Fisher Scientific, Grand Island, NY), then serially diluted in AE and run as a standard for the total bacteria qPCR assay. Based on *V. alginolyticus* strain (ATCC 17749), we assumed our standard contained 11 copy numbers of the target 16S rRNA gene (Stoddard *et al*., 2015). For the *Vibrio*-specific assay, PCR product was generated from genomic DNA using *Vibrio* group specific primers 567F and 680R (Thompson et al. 2004). The PCR amplicon was cleaned (QIAquick PCR purification kit; Qiagen) and inserted into a PCR-4 vector and cloned into *E. coli* using a TA-TOPO kit (Life Technologies Grand Isle, NY). The plasmid was extracted (QIAquick Spin Miniprep kit; Qiagen) and the cloned region sequenced to verify the correct insert. The plasmid was linearized with NotI (Roche, Indianapolis, IN) after cleanup (QIAquick PCR purification kit; Qiagen) and was quantified using a Qubit Fluorometer. The linearized standard was serially diluted in AE and run with each *Vibrio*-specific qPCR assay.

Top-Down Effects from Grazer removal

An experiment was conducted to test if exposure to triclosan was removing top-down predation due to toxic effects on bacterial grazers (Gasol et al., 2002). To test this, an additional set of microcosms was included during experimentation at Clam Bank Landing (North Inlet, Georgetown, SC) with the same treatments: no triclosan added (noadd); 0.05% ethanol (solvent); low triclosan (lowt); high triclosan (hight). Microcosms were processed in the same manner as all others except for a removal of grazers by pre-filtering water (5 μm pore-size) and termed as the no-grazer treatment. Microcosms from Clam Bank Landing with grazers (n = 3) were compared against microcosms with no grazers (n = 2). Results of a full factorial ANOVA indicate main effects of triclosan treatments ($F_{3,12} = 71.7$, $p < 0.0001$), grazer treatments ($F_{1,12} = 24.04$, $p = 0.004$), and the interaction of treatments ($F_{3,12} = 26.44$, $p < 0.0001$) were statistically significant ($\alpha = 0.05$) and accounted for 54.16%, 6.05%, and 19.97% of the variation, respectively. *Vibrio* proportional growth did not appear to be impacted by top-down effects due to triclosan toxicity to grazers, as high triclosan treatments with grazers had higher proportional growth than no grazer treatments (Figure S1), indicating pre-filtering likely removed particle-associated *Vibrio*.

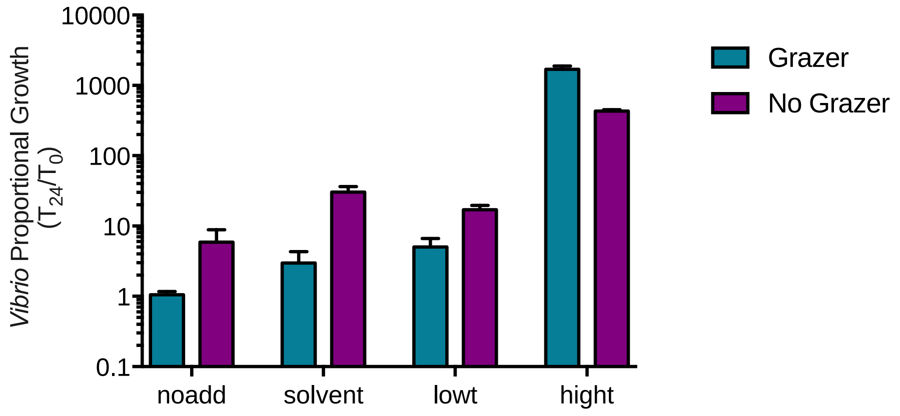

Figure S1. Top-Down Effects of Grazer Removal.

Mean Triclosan Concentrations for Glass vs. Plastic Experiment

An experiment was conducted to test if plastic had an effect on measurements of triclosan concentration during dosing experiments or storage. Treatments included 1 L glass and 1 L plastic bottles filled with 30 ppt salinity Instant Ocean (Spectrum Brands, Inc.) amended with either the high dose or low dose of triclosan. These mock microcosms were mixed and decanted into 50 mL tubes (as done in the main experiment) that were made from either glass or plastic. In total, 4 scenarios were tested at both the low and high concentrations of triclosan with final treatments including: glass to glass (n = 2), plastic to plastic (n = 3), plastic to glass (n = 3), glass to plastic (n = 3). Samples were held in the final 50 ml vessels at –20°C for ~ 4 months. All concentrations of triclosan were measured via LC-MS/MS in the

same manner as described in the methods section. Triclosan concentrations passed normality testing and were subjected to one-way ANOVA ($\alpha = 0.05$) separately for the high dose and low dose sample set in Graph Pad Prism. ANOVA results indicate main effects of treatment were not significant for high triclosan ($F_{3,7} = 0.3603$, $p = 0.7838$) nor low triclosan ($F_{3,7} = 1.22$, $p = 0.3704$). Results suggested that use of plastic in experiments had no significant effect on triclosan dosing or measurements.

Table S1. Mean (and std error, SEM) Triclosan Concentrations for Glass vs. Plastic.

	High dose (μg L^{-1})	SEM	Low dose (μg L^{-1})	SEM
Glass to glass	5,918	93	5.399	0.841
Plastic to glass	7,458	692	7.351	0.714
Plastic to plastic	6,440	1,236	6.926	1.143
Glass to Plastic	7,103	947	6.103	1.049

Table S2. Barcodes for forward and reverse primers used for 16S rDNA sample tagging.

**Sample Name**	**R-primer barcode**	**F-primer barcode**
LK1_T1A_1	CGTAGCAT	AACCAACC
LK10_HT1_1	CGTAGCAT	CGTTCGTT
LK11_HT2_1	CGTAGCAT	GCAAGCAA
LK12_HT3_1	CGTAGCAT	TTCGTTCG
LK13_T1A_2	TTCGTTCG	AACCAACC
LK14_T1B_2	TTCGTTCG	CCAACCAA
LK15_T1C_2	TTCGTTCG	GGTTGGTT
LK16_NA1_2	CCAACGTA	CCATCCTA
LK17_NA2_2	TTCGTTCG	AGTCGACT
LK18_NA3_2	TTCGTTCG	CCATCCTA
LK19_LT1_2	TTCGTTCG	GTCAAGAG
LK2_T1B_1	CGTAGCAT	CCAACCAA
LK20_LT2_2	CCAACGTA	TAGGTTGC
LK21_LT3_2	TTCGTTCG	AAGCAAGC
LK22_HT1_2	TTCGTTCG	CGTTCGTT
LK23_HT2_2	TTCGTTCG	GCAAGCAA
LK24_HT3_2	TTCGTTCG	TTCGTTCG
LK3_T1C_1	CGTAGCAT	GGTTGGTT
LK4_NA1_1	CCAACGTA	AGTCGACT
LK5_NA2_1	CGTAGCAT	AGTCGACT
LK6_NA3_1	CGTAGCAT	CCATCCTA
LK7_LT1_1	CGTAGCAT	GTCAAGAG
LK8_LT2_1	CCAACGTA	GTCAAGAG
LK9_LT3_1	CGTAGCAT	AAGCAAGC

Table S3.1. Looe Key Reef: *Vibrio* spp. concentrations in natural seawater microcosms.

T_0 mean CFU mL^{-1} (n = 3) is 64 CFU mL^{-1}.

Treatment	T_{24} (CFU mL^{-1})	T_{24}/mean T_0	Mean T_{24}/T_0 (n = 3)
No Addition	67	1.04	4.81
	640	10.00	
	217	3.39	
Solvent Control	90	1.41	3.57
	397	6.20	
	200	3.12	
Low Triclosan	203	3.17	1.79
	67	1.05	
	73	1.14	
High Triclosan	5090	79.53	68.6
	5060	79.06	
	3023	47.23	

Table S3.2. Doctors Arm Canal: *Vibrio* spp. concentrations in natural seawater microcosms.

T_0 mean CFU mL^{-1} (n = 3) is 2000 CFU mL^{-1}.

Treatment	T_{24} (CFU mL^{-1})	T_{24}/mean T_0	Mean T_{24}/T_0 (n = 3)
No addition	3500	1.75	1.65
	2067	1.03	
	4333	2.17	
Solvent control	3667	1.83	1.91
	3867	1.93	
	3933	1.97	
Low triclosan	3400	1.70	1.89
	3400	1.70	
	4533	2.27	
High triclosan	1317333	658.67	540.22
	972000	486.00	
	952000	476.00	

Table S3.3. Clam Bank Landing: *Vibrio* spp. concentrations in natural seawater microcosms.

T_0 mean CFU mL^{-1} (n = 3) is 134 CFU mL^{-1}.

Treatment	T_{24} (CFU mL^{-1})	T_{24}/mean T_0	Mean T_{24}/T_0 (n = 3)
No addition	150	1.12	1.05
	110	0.82	
	163	1.21	
Solvent control	223	1.66	2.98
	757	5.65	
	217	1.62	
Low triclosan	390	2.91	5.06
	537	4.00	
	1107	8.26	
High triclosan	279467	2085.57	1701.50
	198133	1478.60	
	206400	1540.30	

Table S4. qPCR cell equivalents per mL for calculation of *Vibrio* abundance index (VAI) (*Vibrio* CE mL^{-1}/total bacterial CE mL^{-1}) for Looe Key experiment.

Treatment	*Vibrio* CE mL^{-1}	total bacterial CE mL^{-1}	VAI	Mean VAI (n = 3)
Time zero	70.9	1.93×10^4	0.0037	0.0036
	4.4	1.21×10^3	0.0036	
	68.5	1.91×10^4	0.0036	
No addition (24 h)	59.9	4.80×10^4	0.0012	0.0013
	165	7.11×10^4	0.0023	
	12.1	3.94×10^4	0.0003	
Low triclosan (24 h)	71.2	3.35×10^4	0.0021	0.0018
	76.7	4.41×10^4	0.0017	
	50.6	3.43×10^4	0.0015	
High triclosan (24 h)	2.58×10^3	1.44×10^5	0.0179	0.0157
	5.77×10^3	2.54×10^5	0.0228	
	1.87×10^3	2.96×10^5	0.0063	

Table S5. Permanova table for the analysis of the weighted UniFrac distance matrix to test the main effects of triclosan treatment on natural seawater bacterial communities.

<table>
<tr><td colspan="7">compare_categories.py --method adonis -i weighted_unifrac_dm.txt -m MappingFileR.txt -c Treatment -o adonisTri_out -n 999</td></tr>
<tr><td></td><td>Df</td><td>SumsOfSqs</td><td>MeanSqs</td><td>F.Model</td><td>R2</td><td>Pr(>F)</td></tr>
<tr><td>Triclosan treatment</td><td>3</td><td>0.3838</td><td>0.1279</td><td>7.9603</td><td>0.7491</td><td>0.001 ***</td></tr>
<tr><td>Residuals</td><td>8</td><td>0.1286</td><td>0.0161</td><td></td><td>0.2509</td><td></td></tr>
<tr><td>Total</td><td>11</td><td>0.5123</td><td></td><td></td><td>1.0000</td><td></td></tr>
<tr><td colspan="7">Signif. Codes: 0 ‘***’ 0.001 ‘**’ 0.01 ‘*’ 0.05 ‘.’ 0.1 ‘ ‘ 1</td></tr>
</table>

Table S6. ANOVA statistics for changes in relative abundance of 18 bacterial families in Looe Key Reef triclosan microcosms.

If differences by ANOVA were determined to be statistically significant, Tukey multiple comparisons test was run comparing treatments (noadd, lowt, hight) to relative abundance at time zero (zero).

	ANOVA		Tukey Multiple Comparisons p-values		
**Bacterial Family**	$F_{3,8}=$	**p-value**	**zero v noadd**	**zero v lowt**	**zero v hight**
Alteromonadaceae	58.72	< 0.0001	0.4562	0.2779	<0.0001
Bradyrhizobiaceae	3.801	0.0581	-	-	-
Colwelliaceae	35.22	< 0.0001	0.9999	0.9751	0.0001
Cryomorphaceae	27.36	0.0001	0.1178	0.3202	0.0001
Flavobacteriaceae	33.47	< 0.0001	0.9992	0.8447	0.0001
Halomonadaceae	10.44	0.0039	0.9972	0.8046	0.0122
Oceanospirillaceae	59.8	< 0.0001	0.6576	0.6466	< 0.0001
OCS155	2.433	0.14	-	-	-
OM60	43.35	< 0.0001	0.0046	0.0002	0.1545
Pelagibacteraceae	7.45	0.0105	0.5474	0.8998	0.0101
Pseudoalteromonadaceae	82.12	< 0.0001	0.1177	0.4241	< 0.0001
Puniceicoccaceae	12.47	0.0022	0.2636	0.7945	0.0066
Rhodobacteraceae	30.95	< 0.0001	0.001	0.9852	0.0849
Saprospiraceae	8.731	0.0066	0.3409	>0.9999	0.0485
Sphingobacteriales; other	14.26	0.0014	0.3269	0.8177	0.0038
Synechococcaceae	1.435	0.3028	-	-	-
Verrucomicrobiaceae	5.544	0.0235	0.9965	0.5763	0.1058
Vibrionaceae	17.65	0.0007	0.9925	0.9783	0.002
